# A Patchier Picture Still: Biases, Beliefs and Overlap on the Inferential Continuum

**DOI:** 10.1007/s11406-017-9881-5

**Published:** 2017-08-04

**Authors:** Sophie Stammers

**Affiliations:** Philosophy Department, ERI Building, University of Birmingham, Edgbaston, Birmingham, B15 2TT UK

**Keywords:** Implicit attitudes, implicit bias, attitude structure, evidence, inference

## Abstract

It has been proposed that, whilst implicit attitudes, alike beliefs, are propositionally structured (Mandelbaum *Noûs, 50*(3), 629–658, 2016), the former respond to evidence and modulate other attitudes in a fragmented manner, and so constitute a sui generis class, the “patchy endorsements” (Levy *Noûs, 49*(4), 800–823, 2015). In the following, I demonstrate that the patchy endorsements theorist is committed to the truth of two claims: (i) no implicit attitude is responsive to content to the same extent as any belief; and (ii) there is a significant gap between the most responsive implicit attitude and the least responsive belief. I argue that both (i) and (ii) fail to hold. Many implicit attitudes respond to evidence and modulate other attitudes. Meanwhile, at least some ordinary beliefs exhibit *lower* evidence-responsiveness and inferential efficacy than at least some implicit attitudes, defeating (i) and (ii). A better interpretation is that attitudes may be ordered along a continuum according to their responsiveness to content. At one extreme end, we find attitudes usually identified as implicit, and at the other, attitudes usually identified as beliefs, but in the middle, there is an area of overlap. I consider the consequences of the continuum view for existing folk psychological concepts.

## Introduction

People who profess to believing in egalitarianism nevertheless make choices or perform actions which disfavour members of (often stigmatized) social groups. For instance, people perform more quickly and accurately on a range of time-pressured tasks when classifying concepts in accordance with a social stereotype compared to when classifying concepts counter to a social stereotype.^1^ Researchers observe a relationship between results on these tasks and real-world discriminatory behaviours: greater bias on matching tasks correlates with greater expression of subtly hostile behaviour (in body language and demeanour, for instance) towards members of already marginalised racial groups in certain social interactions; and also correlates with preferential treatment of certain social groups in more deliberative scenarios, such as when evaluating C.V.s, hiring candidates or prescribing medication.^2^ These findings have been explained by appeal to the notion that people harbour *implicitly* biased attitudes, attitudes which are importantly different to individuals’ considered beliefs and commitments, and, given their ubiquity, it is highly likely that these implicit attitudes play a role in perpetuating inequalities.

Exactly what is implicit about implicit attitudes is the subject of much debate. Some philosophers embrace “dual-process” models on which implicit attitudes have a number of properties which distinguish them from their explicit counterparts. On these models, implicit attitudes, which inhabit “System 1”, are (*a*) unconscious; (*b*) associatively structured; (*c*) automatically activated, and cannot be intentionally inhibited; and (*d*) not avowed. Meanwhile, explicit attitudes inhabit “System 2”, and are (*a* ^1^) conscious; (*b* ^1^) propositionally structured, (*c* ^1^) deliberatively controlled; and (*d* ^1^) avowed (e.g. Evans 2008; Frankish 2010). Implicit attitudes are often measured by employing a time pressured concept classification task, as mentioned above. Perhaps the most well-known of these is the implicit association test (IAT).^3^ Explicit attitudes are usually measured by simply asking people what they believe, or what they feel.

For many, the dual-process model has explanatory value when considering the results discussed at the start of this section, because it provides a framework to account for how people who profess to having egalitarian commitments exhibit prejudicial behaviour. Accordingly, an individual’s professed commitments, which are conscious and considered, are a result of explicit processing, whilst their prejudicial behaviour, which is supposedly non-conscious and automatic, is the output of the implicit system.

The precise nature of the distinction between the implicit and the explicit is up for philosophical discussion in various ways, including, for instance: (1) whether *a*…*d* are necessary and/or sufficient features of implicit attitudes, and ditto for *a* ^1^…*d* ^1^ in the case of explicit attitudes; (2) whether implicit attitudes are a sui generis class, or whether the distinction between them and more familiar attitudes such as beliefs is more graduated; (3) whether these features are *stable* attributes of attitudes; and even (4) whether “implicit” and “explicit” pick out distinctive features at all. Questions 1–4 also interact. For instance, implicit attitudes might form a sui generis class (as in 2) because they have features *a*…*d* necessarily (as in 1). Or, they might form a sui generis class, because to be designated implicit is to fulfil a homeostatic property cluster, whereby an attitude has a sufficient number of features *a…d* without having any particular feature necessarily.^4^ So, much is open to philosophical debate.

Recently, prominent philosophical discussion of the nature of implicit attitudes has focused on *b* and *b* ^1^, the associative-propositional distinction, and it is with this discussion (and its implications) that my paper is primarily concerned. Dominant psychological accounts (to be reviewed in more detail in section 2) hold that explicit attitudes are structured propositions, processed in accordance with their semantic content, whilst implicit attitudes are mere associations between concepts and are processed regardless of propositional information regarding their constituent concepts (e.g. Gawronski and Bodenhausen 2014). Philosopher Eric Mandelbaum, however, has contested the associative model of implicit attitudes in a recent paper (2016). I summarise Mandelbaum’s account and supporting evidence in section 3. Mandelbaum presents evidence which demonstrates that implicit social attitudes often update in light of propositional instruction, which is only possible if they are structured propositions. On the taxonomy presented above, this thesis constitutes a challenge to type-1 discussions which hold that implicit attitudes are necessarily associative, because it shows that many implicit attitudes are in fact propositional in structure. For Mandelbaum, this puts implicit attitudes on a par with beliefs, as regards their structure (but not necessarily as regards other features – Mandelbaum still holds that implicit attitudes are unconscious, for instance). Still, if correct, this argument shows that implicit and explicit attitudes do not instantiate as many genuine distinctions as dual-systems theorists would have it.

Mandelbaum’s proposal that implicit attitudes are (standardly unconscious) beliefs, has been challenged by Neil Levy (2015). Levy accepts that implicit attitudes and beliefs may have their propositional structure in common, but argues that the former exhibit markedly different *responsiveness to content*: in particular, implicit attitudes respond to evidence and modulate other attitudes in a far patchier and more fragmented manner than beliefs. For Levy, the difference is sufficient for implicit attitudes to be considered a sui generis class – the “patchy endorsements” – an argument that is the focus of section 4. If successful, then this proposal nullifies the disruption that Mandelbaum’s account does to (the b/b^1^ component of) the dual-systems account: a distinction is reinstated, but this time, rather than applying as regards the attitudes’ structure, it holds as regards their *responsiveness to content*.

In this paper, I show that the patchy endorsements account is unsuccessful at distinguishing implicit attitudes from beliefs on the basis of the limited content-responsiveness of the former. Levy’s account comprises arguments for there being both a distinction in kind and a distinction in degree in the content-responsiveness of implicit attitudes and beliefs. I argue that the distinction in kind cannot be upheld (3.1), but that the distinction in degree carries force (3.2). I demonstrate that, in order to maintain this distinction, the patchy endorsements theorist is committed to the truth of two claims: (i) that implicit attitudes and beliefs inhabit a continuum as regards the breadth of the content-driven processing that they exhibit, but do not overlap on this continuum; and (ii) that there is a sufficient gap between the last implicit attitude and the first belief on the continuum to uphold that the former constitute a sui generis class. In section 5, I argue that both (i) and (ii) fail to hold. I demonstrate that a variety of beliefs exhibit more limited content-driven processing than at least some implicit attitudes. By dual-system theory’s own lights, these states count as explicit beliefs in other relevant respects because they are conscious, deliberative and avowed. As such, at least some beliefs are *as “patchy”* as some implicit attitudes, and both (i) and (ii) are false.

I propose that a better interpretation of the evidence is that attitudes, both implicit and explicit, are ordered along a continuum as regards the breadth of content-driven processes in which they feature, and upon which no principled sui generis class – as regards content-responsiveness – is identifiable. At one extreme end, we find only attitudes that, in other relevant respects (*a, c* and *d*), are implicit, and at the other, only attitudes such as beliefs (with properties *a* ^*1*^ *, c* ^*1*^ and *d* ^*1*^), but in the middle, there is an area of overlap of both beliefs *and* implicit attitudes. That we find this overlap means we’ve been assigning a category distinction, “patchiness” vs. “inferential promiscuity”, where no such robust distinction really exists.

Clarity on the structure and functional characteristics such as content-responsiveness of implicit biases will clearly play a role in informing strategies to mitigate bias. But it also furthers the conceptual project of better understanding the nature of these attitudes and the relationship in which they stand to more established folk psychological concepts. Understanding of the characteristics of implicit attitudes dictates how we carve up the mental landscape – if it turns out that “implicit” and “explicit” designate regions in which an attitude is likely to be found on a continuum, rather than picking out a distinctive (set of) feature(s) then sui generis class claims do not carve psychological reality at its joints.

In arguing for the claim that patchiness vs inferential promiscuity is not a sufficiently robust distinction to uphold a sui generis class, I’ve not engaged with other features of attitudes, such as consciousness, control and avowal. For the purposes of this argument, I follow dual-systems theorists in holding that distinctions *a*/*a* ^*1*^ *, c/c* ^*1*^ and *d*/*d* ^*1*^ characterise implicit and explicit attitudes. However, full enquiries into the robustness of each of these distinctions will be important for providing satisfactory answers to questions 1–4. In the final section 6, I review work already undertaken on these features as a way to distinguish implicit from explicit attitudes; outline why one may be able to build similar cases for the existence of overlap between implicit and explicit attitudes on the relevant continuums; and recommend potential avenues for future research on the metaphysics of implicit attitudes.

## Two Structural Hypotheses

On a notable psychological model of the mind, there are two kinds of mental structures: propositions and associations (Sloman 1996; Smith and Decoster 2000; Strack and Deutsch 2004; Rydell and McConnell 2006; Gawronski and Bodenhausen 2006, 2011, 2014). Propositions encode information about the nature of the relationship that holds between their constituent concepts. For instance, the proposition ‘Dan loves George’ specifies the relationship that Dan bears to George, that of loving. Propositions may figure in inferential transitions in line with this encoded information. For instance, from ‘Dan loves George’ and ‘Dan has bought a birthday present for the only person that he loves’ it may be inferred that ‘Dan has bought a birthday present for George’.

Associations encode the extent to which an individual has experienced their constituent concepts in spatiotemporal contiguity. Associative mental structures were posited following evidence which demonstrates that the activation of one concept primes a set of other nearby concepts which are then apt to feature in processing. For example, individuals are quicker to recognise ‘butter’ as an English word when first presented with the word ‘bread’, compared to when they were first presented with a word that is unrelated to ‘butter’, such as ‘window’ or ‘doctor’ (Meyer and Schvaneveldt 1971). Assuming that bread and butter occur contiguously in the subjects’ environment more often than butter and windows, researchers interpret these results to show that subjects consequently develop a stronger associative link concerning the former pairing of concepts than the latter. When one concept is activated, other concepts which have a strong associative link with the first become primed for activation, making them more accessible to mental processing than those less closely associated. In contrast to propositions, associations do not encode information about how else the constituent concepts may be related further to their spatiotemporal contiguity, and so from a mere association between Dan and George, the addition of ‘Dan has bought a birthday present for the only person that he loves’ does not yield any inferential results.

Propositions may be learned in a single instance, such as witnessing Dan confess his love for George, or being told by a reliable source that Dan loves George. They may also be learned as a result of witnessing the co-occurrence of two objects multiple times (de Houwer 2014, 344; Gawronski and Bodenhausen 2014, 453).^5^ The hypothesis regarding how associations are modified is symmetrical to that regarding how they are formed: “repeated co-occurrences in the environment may create new associative links between concepts in memory,” (Gawronski and Bodenhausen 2014, 454). As such, an already encoded association between concepts A and B may only be altered through a process of extinction or counter-conditioning: Either A is presented many times in absence of B (extinction), or with an alternative concept C (counter-conditioning).^6^ According to the associative model, a single propositional instruction or inference cannot modify an association (Gawronski and Bodenhausen 2014, 455). This feature yields a test for whether a structure is truly associative. If a structure is modified by a single propositional instruction, then the structure in question is not associative. As Mandelbaum notes, if any implicit attitudes may be shown to update in light of propositional information, then this is evidence that at least some implicit attitudes are in fact propositional rather than associative structures (2016, 637).

## Modification of Implicit Attitudes by Propositional Information

There is evidence that appears to show exactly that – implicit attitudes are sometimes modified in accordance with propositional information. A thorough and illuminating exposition of much of this evidence is given by Mandelbaum (2016, §4), which I paraphrase below. De Houwer (2014) also presents a range of relevant findings regarding both implicit social attitudes and non-social attitudes, which I also include.

### Modification by Strength of Argument

Strength of argument can affect the strength of an implicit bias. Briñol et al. (2008 in Mandelbaum 2016) demonstrate that subjects who are presented with a strong argument for hiring an African American professor (citing their academic merits) exhibit less implicit bias on a subsequent IAT than that exhibited by those presented with a weak argument (citing the benefit to the image of the institution). According to the interpretation on which implicit biases are associative, just the mention of the term ‘African American’ should activate negative implicit associations which could then be observed on a subsequent IAT. Both the strong and the weak argument contained the same number of mentions of the term ‘African American professors’. So, if associations guide IAT responses, then we would expect there to be no significant difference between the performance of the two groups. But this was not what was observed – those who read a strong argument for hiring African American professors exhibited less anti-African American bias on the IAT. All else being controlled for, Briñol et al. (2008) concluded that strength of argument is the variable that accounts for the resulting difference in the level of implicit bias across conditions, indicating that these biases have a propositional structure. This effect has also been observed in non-social attitudes: reading an argument about the positive features of consuming vegetables modifies existing implicit evaluations in favour of eating vegetables (Horcajo et al.^2010^).

### Modification by Relational Information

When reasoning, we tend to evaluate our enemy’s enemy as a friend (see Heider 1958; Aronson and Cope 1968; as cited in Mandelbaum 2016). As discussed in section 2, associative processes are blind to the propositional notion of double-negation elimination. Instead, they process valences individually: a negated negative valence is processed as a negative valence. So, if implicit like/dislike preferences are associative, then an enemy’s enemy will inherit a negative valence, on account of being associated with two negative items.

However, implicit preferences which are sensitive to semantic content of enemy-friend relationships have been observed. Gawronski *et al*. (2005; in Mandelbaum 2016) presented subjects with a series of photos of unfamiliar people (the ‘CS1s’) which were coupled consistently with either positively or negatively valenced concepts. Experimenters then presented subjects with a second series of photos of different people (the ‘CS2s’), as well as information on whether the CS2s were liked or disliked by the CS1s. The associative theory predicts negative attitudes towards the CS2s because participants encounter the CS2s contiguously with the negatively valenced CS1s, thus activating another negative valence (Mandelbaum 2016, 639). But the implicit attitude measure produced results which indicated that quite the opposite occurred. Participants exhibited implicit *preferences* for the CS2s who were disliked by negatively valenced CS1s – preferences for the enemies of their enemies. These results imply that implicit preferences are sensitive to the logic of double negation elimination, and are neither predictable nor explicable by a theory on which implicit like/dislike preferences are associative (Mandelbaum 2016, 640).

Peters and Gawronski (2011) conducted a later study in a similar paradigm, (in De Houwer 2014). They presented participants with pictures of four unknown people alongside a series of personality traits. Half of the pictures were presented alongside positive traits; the others presented alongside negative traits. Participants were informed that only some of the people were paired with words that described their actual traits, others were paired with opposing words. Participants then underwent an implicit attitude measure regarding their attitudes towards the pictures. Results on implicit measures demonstrated that participants evaluated the person paired with words which (falsely) described them positively *less favourably* than they evaluated the person paired words which (truly) described them negatively. Implicit evaluations distinguished truth from positivity and negation from negativity.

These findings demonstrate that at least some implicit social preferences and evaluations are modulated by propositional information regarding the relationship between concepts, leaving the associative theory of implicit evaluations unable to explain these results.

### Modification by Abstract Propositional Instruction

Implicit attitudes which are formed as the result of an abstract propositional instruction (requiring participants to assume that a social group possesses some particular characteristic) can be as strong as implicit attitudes formed as the result of extensive associative conditioning. In a study by Gregg et al. (2006; cited in Mandelbaum 2016), half of the participants read a sentence about which of two (fictitious) tribes are peaceful and civilised, and which are savage and barbaric. The other half underwent 240 trials to associatively condition (through contiguous presentation) one tribe with the notions of peace and civilisation, and the other with savagery and barbarism. Those in the propositional instruction condition showed implicit attitudes (as measured by the IAT) which were as strong as those in the associative learning condition. So, being presented with an abstract proposition in which a social group are predicated with a valenced concept produced *as efficacious* a behavioural response on the IAT as extensive associative conditioning. That participants who have seen two concepts in spatiotemporal contiguity only once have as strong an implicit attitude as those who have seen the concepts in spatiotemporal contiguity 240 times is not explainable on the associative hypothesis (Mandelbaum 2016, 644).

Further, additional propositional information modulates these implicit attitudes to a greater extent than further associative conditioning. In a subsequent study, participants in the propositional instruction condition were required to read a sentence in which the adjectives which originally described the first tribe now described the second, and vice versa. Those in the associative condition underwent extensive counter-conditioning trials to switch their associations accordingly. This counter-conditioning failed to have any effect on subjects’ attitudes. However, an IAT of those in the propositional instruction condition revealed that their implicit attitudes *had* in part adjusted in line with the new information, as they were shown to be less extreme than attitudes on the first test, and less extreme than those in the associative condition.

Modification of implicit attitudes by abstract propositional information also occurs in non-social attitudes, as demonstrated by De Houwer (2014) who claims that “a single instruction about the relation between the meaningless words and positive or negative pictures was sufficient to influence the implicit evaluation of the words” on an IAT (2014, 347). This indicates that the underlying attitudes are propositional.

### Evidential Adjustment to Peer Attitudes

Sechrist and Stangor (2001; in Mandelbaum 2016) measured participants’ prejudice via a questionnaire before they took a seating distance test. They demonstrated that participants high in prejudice who were informed that their peers agreed with their racial prejudices sat further from the African American confederate than those informed that their peers disagreed with them. Participants low in prejudice who were informed that their peers agreed with them sat closer to the confederate than those informed that their peers disagreed with them. Mandelbaum holds that the associative hypothesis of implicit bias cannot explain these findings, citing findings which show that peer agreement creates a positive affect, whilst peer disagreement creates a negative affect (Elliot and Devine 1994). Accordingly, an associative hypothesis would predict the opposite findings: Agreement from one’s peer group for high-prejudice participants should create a positive affect, lowering fear-responses and lessening the distance sat from the confederate, whilst disagreement from one’s peer group for high-prejudice participants should create a negative affect, thus increasing seating distance – the reverse of what was in fact observed. But a hypothesis on which implicit biases encode propositional information, and which may be adjusted in light of evidence (albeit evidence of one’s friend’s attitudes) does predict the results observed (Mandelbaum 2016, 642–3).

Mandelbaum does not conclude from this evidence that implicit attitudes have all of the relevant properties that one might maintain are necessary for prototypical beliefs (such as *a* ^1^, *c* ^1^ and *d* ^1^ as discussed in the introduction, for instance). Rather, his claim is that implicit biases have the requisite *structural* properties of beliefs. If true, then the *b*/*b* ^1^ distinction does not hold, and things are not as clear-cut as the dual-systems theorist would have it.

## Implicit Biases as Patchy Endorsements

Neil Levy (2015) has recently critiqued Mandelbaum’s (2016) view. Levy acknowledges that at least some of the evidence summarised above demonstrates that implicit biases, and implicit attitudes more generally are propositionally structured, because they have been shown to respond to semantic content, and to feature in content-driven transitions. But Levy does not accept Mandelbaum’s conclusion wholesale, arguing that beliefs exhibit these characteristics *systematically* (2015, 801). As such, he argues that implicit attitudes are a sui generis class, the “patchy endorsements” – attitudes that sometimes exhibit some of the content-responsiveness of beliefs, but not all of the time. Consequently, something in place of the *b*/*b* ^1^ distinction may yet hold, this time as regards content-responsiveness. For Levy, this result is significant not just for conceptual debates as regards how we carve up the psychological landscape, but also as regards the moral responses that track features of our psychological distinctions. In particular, a person acting on a biased *belief* invites a particular moral response, a response that Levy thinks should be reined in if it turns out that person in question acted only on a biased patchy endorsement (2015, 816–817).

The content-responsiveness that, according to Levy, distinguishes beliefs from implicit attitudes manifests in two ways. Firstly, beliefs are “inferentially promiscuous” in that they tend to cause the update of other propositional attitudes, in “normatively respectable” ways (2015, 805). Levy does not define what is meant by a “normatively respectable inference”, but gives an example pertaining to interaction with other states with relevant content in line with some generally accepted standards of reasoning: The belief that it is raining, for example, will interact appropriately with the desire to stay dry, the belief that roads can be dangerous when wet, and other attitudes concerning water, and so on (Levy 2015, 805). Secondly, beliefs are responsive to evidence, and can be expected to update when appropriate evidence is available. These two characteristics are interrelated for Levy: “Inferential promiscuity and responsiveness to evidence are two sides of the same coin: beliefs are inferentially promiscuous, causing the update of other beliefs, because beliefs are responsive to evidence” (ibid). For Levy, promiscuity of these two characteristics is *necessary* for belief (without yet being sufficient, but note that properties *a* ^1^, *c* ^1^ and *d* ^1^ are not under discussion – the patchy endorsements argument is focused on content-responsiveness).

Whilst implicit attitudes may feature in content-driven processing, “they are not beliefs because their responsiveness to content is fragmented, and often the responsiveness they exhibit is of the wrong sort to count as genuine inference” (2015: 801). There are two distinct claims here, which are exemplified in the paper more broadly, and which I will address in the following two subsections. There is a claim about the *degree* to which implicit attitudes feature in content-driven transitions: implicit attitudes interact with content in a limited manner, whilst beliefs interact promiscuously. There is also a claim about the *kind* of transitions that implicit attitudes (fail to) feature in, and this, according to Levy, rules out implicit attitudes as being properly considered as featuring in inference all. The latter is a stronger claim, so let’s address it first.

### A Difference in Kind

To maintain a difference in kind between the content-responsiveness of implicit attitudes and that of beliefs it must be the case that all beliefs have some kind of responsiveness – to whatever degree – that *all* implicit attitudes lack (or vice versa). I maintain that the evidence that Levy supplies on the way that implicit attitudes are processed does not give us sufficient reason to buy into this stronger distinction, the distinction in *kind*. Even though he presents evidence that some implicit attitudes fail to respond to some kinds of content, further evidence demonstrates that other implicit attitudes do respond to such content, as I will now discuss.

Insensitivity to logic, specifically the logic of negation, is one area where, according to Levy, implicit attitudes fail to exhibit the right *kind* of responsiveness to count as doxastic (Levy 2015, 816). Indeed, a number of studies do demonstrate that at least *some* implicit attitudes are blind to negation, at least in the absence of specific training (Deutsch 2006; Hasson and Glucksberg 2006). However, the difference in kind claim is not supported, because further studies suggest that other implicit attitudes do respond to negation: When Armstrong and Dienes (2013) presented participants with unconscious primes that instructed them to either pick or to not pick a word, participants choose in line with the instruction (including when it contained a negation) at a higher-than-chance rate. Because the primes were subliminally presented, the attitudes governing participants’ reactions were neither conscious, nor amenable to deliberative control. Nor were they avowed by participants. As such, these attitudes have features *a, c,* and *d,* features by which dual-systems theorists’ own lights qualify them as implicit. Participants do not do much better than chance, but that there is a relationship of significance between instruction and picking at all demonstrates that, at least *sometimes,* implicit attitudes may be formed in a manner sensitive to the logic of negation, and may guide successful behaviour. Indeed, that implicit attitudes respond to enemy-of-my-enemy-is-my-friend logic looks like evidence that they are sensitive to some species of double negation elimination (Gawronski et al. 2005; in Mandelbaum 2016).^7^

It’s salient that a similar picture emerges elsewhere concerning the responsiveness of implicit attitudes to logical and semantic information. People may unconsciously differentiate congruent from incongruent subliminally presented symbolic representations (discerning a difference when ‘a’ and ‘D’ are subliminally present, but not when ‘a’ and ‘A’ are) entailing that the meaning of symbolic representations may be processed unconsciously (van Opstal et al. 2010). Further, simple addition can occur unconsciously (Ric and Muller 2012), whilst subliminal presentation of “2 × 3” enables participants to pronounce “six” more quickly than other numbers (Garcia-Orza et al. 2009) and subliminal presentation of “9–4-3” enables participants to pronounce “two” more quickly than other numbers (Sklar et al. 2012). The attitude in question may be fleeting (“the answer to *x-*minus*-y-*minus*-z* is *w*”) but it exists long enough to manifest in behaviour. Once again, the subliminally presented primes entail that that the attitudes guiding behaviour have features *a, c* and *d*. These attitudes are, in other important respects, implicit. So, sensitivity to negation and other logical rules does not mark a difference in kind between the content-responsiveness of implicit attitudes and beliefs, just one of degree. We should perhaps not find this surprising: once implicit attitudes are modelled as fundamentally propositional, we might expect to find evidence of content-driven processing in many scenarios, even if this kind of processing is usually somewhat limited.

Levy makes a suggestion that could threaten this interpretation, saying that “[r]ather than the semantic content of the implicit attitude playing an inferential role, the attitude plays the role of disposing the person toward some options and away from others” (2015, 814). It could be that this occurs for some transitions involving implicit attitudes. But the dispositions would have to be *extremely* fine-grained to account for all of the above evidence, particularly that from Sklar et al. (2012). This interpretation of their study entails that there would be distinct dispositions which prescribe responses to all the possible three-term math operations, for example. That’s as many as a thousand distinct dispositions (presuming processes are insensitive to commutability, a content-driven notion) attached to just 10 symbols. Meanwhile, no disposition should arise that delivers a wrong answer to a three-term operation. Why should dispositions arise like this? It’s hard to see what story could be told to account for this many dispositions without resorting to content-sensitivity.^8^

Findings in clinical settings also demonstrate evidence-sensitive implicit attitudes which go on to guide behaviour after corresponding explicit attitudes have decayed. Edouard Claparède describes a patient in his care with Korsakoff’s syndrome (1951). Confabulation, in which a person gives an ill-grounded narrative of some occurrence without the intention to deceive, often in the absence of accurate memories of the occurrence in question, is a common feature of Korsakoff’s, and one seen in Claparède’s patient. In an (undoubtably cruel) attempt to determine the limits of his patient’s memory, Claparède pricked her hand with a pin held between two fingers. This episode was apparently swiftly forgotten by the patient. However, when he reached for her hand again, she drew it away, but gave reasons other than that he had recently pricked her with a pin in explanation. As such, the attitude driving the withdrawal of her hand was neither conscious nor avowed. Uleman et al. (2005), who discuss the case, suggest that we should interpret this as an example of a broadly accurate, yet *implicit* impression that persists when the explicit impression has decayed, and which enables content-driven behaviour (self-protection in the presence of an anticipated pin-prick).

So, to conclude this section, Levy has not provided evidence that all beliefs have content-responsiveness characteristics that all implicit attitudes lack (or vice versa). This means that the proposed distinction must be one of *degree*: it must be cashed out in terms of the breadth of content-driven transitions in which each sort of attitude figures.

### A Difference in Degree

Levy makes a persuasive case for thinking that there is a difference in the breadth of content-driven processes in which each sort of attitude features. For instance, he cites evidence in which implicit attitudes about fictional characters update in line with information given in testimony, but fail to update in line with the instruction that this information is incorrect (Han et al. 2006; in Levy 2015). Further, the fact that egalitarian beliefs persist in people with implicit biases demonstrates that the latter fail to appropriately interact with beliefs, and thus fail to be inferentially promiscuous (Levy 2015, 806). So, the difference in degree claim is persuasive.

What, then, are the core commitments of the patchy endorsements theory? Recall that Levy thinks that patchy endorsements are a sui generis class, as regards their limited content-responsiveness (2015: 800). But if we can’t get any distinctions in *kind* between the content-responsiveness of beliefs compared to that of implicit attitudes, then the proposed distinction needs to be built out of there being a sufficiently large degree of difference in the *extent* to which beliefs exhibit content-responsiveness as compared to implicit attitudes. Levy maintains that it may well be “eliminably vague” as to exactly how content-insensitive an attitude must be to no longer qualify as doxastic (2015: 806). Nevertheless, at some point “sufficient departure from the kind of sensitivity to evidence and aptness for normatively respectable inference we associate with a *bona fide* belief will settle the question” (ibid). If a difference in degree at some point generates a difference in kind, then the patchy endorsements theorist is committed to the following two claims:

- **Ordered continuum claim (OCC)**

Each and every state in the set of beliefs, *b*_1_, *b*_*2*_, *b*_*3*_...*b*_*n*_, is responsive to content more frequently than each and every state in the set of implicit attitudes *ia*_1_, *ia*_*2*_, *ia*_*3*_...*ia*_*n*_.

- **Sufficient gap claim (SGC)**

There is a sufficient gap between the least content-responsive belief, *b*_*L*_, and the most content-responsive implicit attitude, *ia*_*M*_ such that *b*_*L*_ is properly considered a different *kind* of state to *ia*_*M*_.

OCC just specifies the commitment to the difference being one of degree, not kind. The distinction has to lie in the frequency with which attitudes respond to content: for example, some belief, *b*_*23*_ *,* responds to content *c*_*1*_, *c*_*2*_, *c*_*3*_, *c*_*4*_, and *c*_*5*_, whilst some implicit attitude, *ia*_*67*_, responds only to *c*_*1*_ and *c*_*2*_. In other words, whilst *ia*_*67*_ may be somewhat responsive to content, only *b*_*23*_ is promiscuously responsive to content.

OCC is spelled out as claim regarding beliefs’ *satisfaction* of content-responsiveness requirements, but one might wonder whether the patchy endorsements theorist might advocate something a little less committal: that beliefs must only be *subject to* content-responsiveness requirements, without necessarily fulfilling them all of the time. This requires an account of the relevant distinction between an implicit attitude that doesn’t update because it isn’t subject to particular content-responsiveness norms, and a belief that doesn’t update because, whilst it is subject to these norms, it simply isn’t complying today. Such an account can be given by someone who thinks that implicit attitudes are associative, because the relevant distinction is simply that implicit attitudes do not have the requisite structure to update in light of new inferential content. But this strategy isn’t open to the patchy endorsement theorist, who accepts that implicit attitudes, being propositional, *do* have the requisite structure to update in light of new content. So, the patchy endorsements theorist must give the relevant distinction in terms of satisfaction of content-responsiveness requirements, rather than simply being subject to such requirements.

OCC is the minimum commitment for the patchy endorsement theorist to be able to say that all beliefs are responsive to content to a *higher* degree than all implicit attitudes, in that it orders implicit attitudes and beliefs on a continuum of content-responsiveness. It does not, however, determine the distance between the most content-responsive implicit attitude, and the least content-responsive belief. If that distance is relatively small, then one may well wonder why we should draw a line at that point on the continuum and designate everything on one side of it a belief and everything on the other side a member of a sui generis class, the implicit attitudes. The distinction would not then be principled, but arbitrary.

SGC is the stronger commitment which delivers the desired difference in kind, because it accounts for *why* the distinction is drawn where it is. In fact, for patchy endorsement theorists, the gap will be quite noticeable. To account for why a sui generis class, the implicit attitudes, is identifiable on what would otherwise be a continuum of steadily increasing content-responsivness, the extent to which ia_M_ responds to content must be more comparable to the extent to which ia_L_ (the least content-responsive implicit attitude) responds to content than it is to the extent to which b_L_ responds to content. Put simply, the claim is that, as regards content-responsiveness, all beliefs are more like each other than they are like any implicit attitudes, and, likewise, all implicit attitudes are more like each other than they are like any beliefs, justifying the postulation of a sui generis class as regards content-responsiveness.

So, OCC establishes the implicit attitude-belief continuum as regards breadth of content-responsiveness, and SGC specifies the gap between implicit attitudes and beliefs which justifies the sui generis class claim. One might accept OCC, but reject SGC. For example, one might maintain that implicit attitudes and beliefs are ordered along a continuum of responsiveness to content, but that the gap between the most content-responsive implicit attitude and the least content-responsive belief is not sufficient to account for a difference in kind between implicit attitudes and beliefs. However, if we found as much as one belief that was less content-responsive than at least one implicit attitude, then OCC would fail to hold. Such a discovery would entail an area of overlap on the continuum such that we wouldn’t be able to draw even an arbitrary line between the last implicit attitude and the first belief, because this line would have both implicit attitudes and beliefs on either side. If we find this area of overlap, then “implicit” and “explicit” do not designate a clean distinction, at least as regards these attitudes’ content-responsiveness. In section 5, I propose that it is this picture that obtains.

## Overlap on the Continuum

In this section, I argue that it is not the case that implicit attitudes are, by their nature, regularly unresponsive to content. This gives us reason for thinking that, in order to include the most evidence-responsive, inferentially efficacious implicit attitudes, the sui generis class will end up occupying space rather far up the content-responsiveness continuum. I also demonstrate that it is not the case that beliefs are, by their nature, regularly responsive to content, and that a great many beliefs may be subject to encapsulated reasoning and resistant to evidence, persisting even when this is brought to the agent’s attention. Not only does this give us reason to think that implicit attitudes and beliefs are much closer on the content-responsiveness continuum than SGC predicts, we can only account for these results by positing an area of overlap (where some implicit attitudes are found higher up the continuum than some beliefs) thus violating even the weaker OCC. As a result, some beliefs are just as “patchy” as some implicit attitudes, giving us a reason to think that the content-responsiveness continuum does not contain genuine sui generis classes.

It’s not clear that implicit attitudes are, by their nature, inferentially inert. Implicit *biases* regularly interact inferentially with a wide variety of beliefs. For example, an implicit bias regarding the superiority of white men in professional environments is apt to update a number of beliefs with content concerning white men: beliefs about who to hire, beliefs about who to promote, beliefs about who to praise in a meeting, etc. That implicit biases are apt to do this is part of why they’re so concerning. Furthermore, such transitions involving implicit biases would often seem to count as normatively respectable. Even though we might think that implicit biases themselves are false or ill-grounded, once they are part of an individual’s psychological economy, they can figure in normatively respectable transitions. An entrenched implicit bias with the content that men are superior in professional environments is the right kind of thing to lend normative support to the *belief* that one should hire the white man – even if in fact one *shouldn’t* hire the white man. Of course, one might object to this by insisting that normatively respectable transitions can only occur between well-grounded attitudes. But this wouldn’t serve to distinguish the kind of inferences implicit biases are involved in from those involving beliefs, as ill-grounded and false beliefs frequently figure in inferences.

Levy (2015) maintains that when implicit biases modify beliefs in the way described in the previous paragraph, these instances do not really count as inferences proper because they “[ignore] too many other representations which we can justifiably attribute to the person” (2015: 814). He accepts that such instances comprise content-driven transitions between propositional states. But he worries that because the putative inference in the hiring example remains encapsulated from many other attitudes (such as the person’s egalitarian commitments, which do not block this inference from being made) we should not consider this as a case of inference (2015, 814).

But there is trouble here. Attitudes which look much like beliefs are regularly involved in inferences which are encapsulated from a significant subset of an individual’s other beliefs. The forthcoming examples look much like beliefs because they exhibit properties *a* ^*1*^ *, c* ^*1*^ and *d* ^*1*^: they are conscious when they guide behaviour; they guide deliberative behaviour; and the individual is disposed to avow their contents.

Consider David Lewis in the following:

I used to think that Nassau Street ran roughly east-west; that the railroad nearby ran roughly north-south; and that the two were roughly parallel… So each sentence in an inconsistent triple was true according to my beliefs, but not everything was true according to my beliefs. (Lewis 1982, 436)

Lewis might often token two of the above three beliefs, and because any two beliefs in the above set are consistent, they were able to persist and feature in relevant inferences for some time. Examples like this are possible because we often form beliefs without consulting the entirety of our belief set to check for consistency.

Lisa Bortolotti (2009) provides a wealth of evidence showing that people often adopt beliefs with dissonant contents, which implies that beliefs do not always block the acquisition of further beliefs with which they are inconsistent. For example, when given a choice between a ticket in lottery A (high likelihood of winning) or lottery B (low likelihood of winning), people exhibit a preference for a ticket in lottery A over lottery B, but chose to price a ticket for lottery B higher than A. Here, people both value ticket A higher than ticket B, and value ticket B higher than ticket A, generating dissonant beliefs about which ticket is more valuable, but the presence of the first belief does not prevent them from acquiring the second. As Bortolotti demonstrates, people exhibit this kind of inconsistency in a range of other contexts, such as when weighing up the value of a certain period of illness vs. good health (Stalmeier et al. 1997), making policy decisions (Tversky and Thaler 1990), and when assessing medical interventions (Tversky and Kahneman 1981). As with the Lewis example, the attitudes in this paragraph are considered beliefs because they exhibit properties *a* ^*1*^ *, c* ^*1*^ and *d* ^*1*^.

Bortolotti also invokes superstitions that persist in otherwise rational individuals. Consider the case of Nishad who wears a charm because he believes it brings him luck, despite also believing that physical objects have no special luck-bringing qualities (Bortolotti 2009, 93). Again, superstitions are conscious; guide behaviour deliberatively; and are avowed. Bortolotti cites Vyse (1997), who demonstrates that these kinds of cases bear out in reality amongst students, gamblers, entertainment and sporting professionals, police officers and medics. Bortolotti emphasizes that often the people involved in the cases described above do not revise at least one of their dissonant beliefs when made to confront their dissonance. This is true of research participants after debriefing (Lichtenstein and Slovic 1971; Stalmeier et al. 1997), as well as anecdotally – Bortolotti’s own undergraduate students who take some of the above reasoning tests refuse to concede that they should exclude at least one attitude, even after debriefing (Bortolotti 2009, 87).

By Levy’s reasoning, because both Lewis’s inferences, as well as those made by participants in the range of situations described by Bortolotti, ignore the content of other beliefs, they do not meet the necessary content-responsiveness requirements to count as beliefs. But insistence on this principle may well vastly shrink the set of attitudes that intuitively ought to count as beliefs. That dissonant beliefs arise in such a breadth of scenarios – from valuing lottery tickets and various health outcome scenarios to policy, town planning and medical spending decisions – suggests that there is a general deficit in the mechanism for checking beliefs about the value of some outcome for consistency with other relevant beliefs. The deficit in this mechanism means that inconsistency may manifest whenever we form beliefs about the value of outcomes: our beliefs about how good *any* outcome is versus another have the potential to be formed in this way, and to thus exhibit this inconsistency. So, if consistency with all or most other attitudes is necessary for an attitude to be considered a belief, but failure to consistency check is prevalent, then very few attitudes that are alike beliefs in many respects (they exhibit features *a* ^*1*^ *, c* ^*1*^ and *d* ^*1*^) will end up counting as beliefs, and this would be a somewhat counterintuitive result.

Imagine confronting someone who advises you that option A is preferable to option B with “you don’t *really* believe that.” It’s likely they would respond with belief-consonant behaviour, such as reaffirming the content of belief, and providing further reasons for which they believe it (as mentioned in the foregoing paragraph, this was observed by Lichtenstein and Slovic 1971; Stalmeier et al. 1997; and Bortolotti 2009). It is preferable to countenance that attitudes which are conscious, deliberative, sincerely avowed, and defended when questioned are beliefs, even if they are dissonant with other attitudes.

Now we have a situation in which at least some implicit attitudes are involved in content-driven transitions in which they mediate many other attitudes with relevant contents, whilst at least some beliefs fail to update the majority of relevant attitudes. It’s hard to adjudicate exactly where these attitudes will lie on the content-responsiveness continuum, but it seems reasonable to assume that there will be at least some overlap between the implicit attitudes and the beliefs in question: at least some implicit biases interact with many beliefs, whilst superstitions might remain relatively inferentially inert (the belief in the supernatural powers of a lucky charm does not extend to physically similar objects, and does not cause one to doubt that the laws of physics hold elsewhere).

Levy acknowledges Bortolotti’s view that some degree of insensitivity to evidence and low inferential efficacy is characteristic of attitudes that appear in other respects to be belief-like, but suggests that supporters of this view “would surely agree, however, that excessive evidence insensivity and encapsulation blocks the ascription of a correlative belief to an agent” (Levy 2015: 806 *sic*). But exactly *how much* evidence insensitivity and inferential inefficacy a belief will tolerate before it is relegated to sub-doxastic status is exactly what is at issue in the patchy endorsement theorist’s argument. I submit that if we accept Lewis and Bortolotti’s examples of attitudes as beliefs (and I think that we should, for reasons given above), then the degree of inferential efficacy that is necessary for attributing a belief does *not* clearly exceed the degree of inferential efficacy achieved by many implicit attitudes. We have a picture where beliefs and implicit attitudes overlap on the content-responsiveness continuum. This constitutes a challenge to both SGC, the stronger patchy endorsements claim, and OCC, the weaker claim.

Finally, consider the systematic resistance to evidence of at least some *explicit* social prejudices. Not all explicitly believed biases will turn out to be resistant to evidence update. It’s possible that someone who believes that women are bad at maths might modify this belief after meeting a brilliant maths professor, or someone who believes that men are not nurturing enough to raise children, might modify their belief after witnessing a capable and loving single father. But there are plenty of other cases of explicitly biased beliefs that are more recalcitrant than the aforementioned, cases which persist in the face of continued counter-evidence. In fact, some suggest that this very tendency characterises explicit prejudice:

If a person is capable of rectifying his erroneous judgements in the light of new evidence he is not prejudiced. *Prejudgements become prejudices only if they are not reversible when exposed to new knowledge.* A prejudice, unlike a simple misconception, is actively resistant to all evidence that would unseat it. (Allport 1954/2000: 23)

Consider Arpaly’s (2003) example of Soloman. Soloman grows up believing that women are unable to engage in abstract thought, without much evidence to the contrary. But then he attends college, where he witnesses plenty of examples of women engaging in abstract thought. Arpaly says

At the end of his first year as a college student, if Solomon were rational, he would have changed his mind about the aptitude of women for abstract thinking. If at the school year's end Solomon still believed that all women are bad abstract thinkers, his belief would now be not only false but also irrational. He would no longer be simply mistaken, but *prejudiced*. (Arpaly 2003: 104)

Failing to update his belief in light of a year’s worth of evidence is the very thing that renders Solomon’s belief a prejudice*.* In fact, it would seem that we can only explain why explicit prejudices like Soloman’s persist by characterising them as regularly unresponsive to evidence. This is not to say that entrenched prejudices cannot be overcome, but evidence suggests that this process requires some motivation, is typically not immediate and is not usually achieved via a single propositional instruction (Hogan and Mallott 2005; Case 2007).^9^

So, the least evidence-responsive belief has set the bar of responsiveness to evidence—a bar which, for the patchy endorsements theorist, must not be exceeded by *any* implicit attitude—as rather low indeed. It seems entirely possible that a person could remain prejudiced for a lifetime, in which case, this would be an example of a belief which is almost *entirely* unresponsive to evidence. It is unlikely that the most evidence-responsive implicit attitude is still less evidence-responsive than this. As we saw in the above, at least some implicit attitudes are sensitive to semantic content and logical rules, and update in light of propositional instruction. On a neutral notion of what responding to evidence is (and it’s up to patchy endorsement theorists to provide a non-neutral alternative) these implicit attitudes count as evidence-responsive. Once again, we find overlap on the content-responsiveness continuum.

Let us take stock. We saw that the stronger commitment of the patchy endorsements theorist, SGC, does not stand up to scrutiny. There are at least some beliefs which respond to evidence and cause inferential updates in much the same way as at least some implicit attitudes. The weaker commitment of OCC, the ordered continuum claim, may get things broadly right in that there may well be a number of highly content-responsive beliefs and a number of highly content-unresponsive implicit attitudes that occupy the extreme ends of a continuum. But it is not wholly right. In fact, it’s a patchier picture still: at least some implicit attitudes are more content-responsive than at least some beliefs, and so rather than a clear divide between the last implicit attitude and the first belief, we’ll instead find a patch of overlap on the content-responsiveness continuum.

This result not only has theoretical consequences for how we carve up the psychological landscape. It also reopens ethical questions that Levy (2015) considered closed. Recall Levy’s suggestion that moral responses track the metaphysical features of attitudes (2015, 816–817). He suggested that a person acting on a biased belief invites a different moral response to that invited by a person acting on a biased patchy endorsement. If the patchy endorsement in question inhabits the area of the continuum in which beliefs are also to be found, then the moral assessment should also be sensitive to how things stand with these beliefs (at least as regards their content-responsiveness). Whilst other features (awareness, for instance) are likely to be relevant to moral assessment, if lack of responsiveness to content does not exculpate in the case of a biased belief, then it may not exculpate in the case of a biased implicit attitude inhabiting the same region of the continuum.

## Beyond Content-Responsiveness

I this paper, I have established that recent efforts to reinstate a sharp distinction between implicit attitudes and beliefs on the basis that the former necessarily respond to content in a different manner to the latter do not achieve their aim. This argument establishes that when implicit attitudes (demarcated by features *a*, *c*, and *d*) and beliefs (demarcated by *a* ^1^, *c* ^1^, and *d* ^1^) are ordered on a continuum just as regards their content-responsiveness, in the middle of this continuum there is an area of overlap comprising both implicit attitudes and beliefs. For the purposes of this argument, I have been assuming that features *a/a* ^1^, *c/c* ^1^, and *d/d* ^1^ do admit of clean distinctions which enable us to demarcate implicit attitudes from beliefs. So, I have not shown, *all features considered*, that implicit attitudes do not constitute a sui-generis class. Rather, the conclusion is more modest: it is that no distinction as regards content-responsiveness will uphold the sui generis class claim: Contra-Levy (2015), patchiness does not mark out implicit attitudes as a sui generis class. If all implicit attitudes are patchy, then at least some beliefs are patchy too.

It is consistent with what I have presented here that implicit attitudes, qua attitudes that harbour features *a*, *c* and *d,* constitute a sui generis class. It’s simply that content-responsiveness plays no part in this story. In what remains, however, I briefly acknowledge results that may present a challenge to distinctions on the basis of *a/a* ^1^, *c/c* ^1^, and *d/d* ^1^ and indicate how future research could help to illuminate whether these putatively distinguishing features really are up to the job.

### Awareness

Studies demonstrate that some implicit attitudes are sometimes accessible to awareness (​Monteith et al. 2001; Hahn et al. 2014). Jules Holroyd has discussed these results extensively (2015). She maintains that they show us that implicit attitudes are only available to *observational* awareness, in which one infers the content of one’s attitude from one’s behaviour, not to *introspective* awareness, in which one is acquainted more directly with this content through introspection. This amounts to there still being a distinction between the kind of awareness that we have of implicit attitudes as compared with that which we have of beliefs. However, one might take issue with her interpretation of these results. In the Hahn et al. (2014) study, participants are shown to be able to *predict* discrepancies in their behaviour on the IAT before they take the test. One might argue that observational awareness is insufficient for the successful prediction of future behaviour. After all, one cannot observe a behaviour that one has not yet performed. If this is correct, then it might be that one can sometimes be aware of one’s implicit attitudes in just the same way that one is aware of one’s beliefs. It would be premature to affirm that conclusion here, but this possibility may form the basis of future research on awareness of implicit attitudes.

### Control

Jules Holroyd and Daniel Kelly argue that implicit attitudes *can* be intentionally inhibited through “ecological control”, a form of control in which cognition is distributed across environmental artefacts, or achieved through cognitive rehearsal techniques (Holroyd and Kelly 2016). Whilst Holroyd and Kelly concede that beliefs are amenable to a form of direct control that implicit attitudes are not, they do demonstrate that ecological control underlies a “vast swathe of human behaviour and problem-solving” (2016, 123). If one could show that at least some *beliefs* are *only* amenable to ecological control, then the distinction between all implicit attitudes and all beliefs as regards how they are controlled may collapse.

### Avowal

Whilst I have taken participants’ avowal at face value in this paper, I acknowledge that it is difficult to *ensure* that one’s experiment measures genuinely avowed attitudes, especially in the case of social attitudes. This is because avowal can be mediated by what experimental psychologists call “social desirability” concerns (e.g. Holtgraves 2004). These are concerns that one’s attitudes are seen to be in line with social norms. Research demonstrates that people may paint themselves in a better light when answering explicit attitude questionnaires (e.g. Fazio et al. 1995; Nier 2005). If attitude avowal can be modulated by social context, then it may not always be absent in the case of implicit attitudes, and may not serve to distinguish them from beliefs.

These are preliminary considerations, and more extensive research would be required to settle whether awareness, control or avowal distinguish implicit attitudes from beliefs following these considerations. Such research would enable us to provide more substantive answers to questions 1–4 as laid out in the introduction. A similar methodology to that employed here may be used, where attitudes are ordered according to their characteristics, and we look to see if there is any overlap. Further research may demonstrate that robust distinctions remain as regards awareness, control and avowal. Or it may demonstrate that these distinctions give way to further continuums featuring overlap. Perhaps, then, “implicit” and “explicit” designate only regions in which one is *likely* to find the attitude in question, as opposed to where an attitude is guaranteed to lie. Or perhaps we will discover that the features considered in this section are not stable attributes of attitudes themselves, but are determined by incidental features, such as the social context in which we find ourselves. Such research will help to settle the wider question as to whether “implicit” and “explicit” in general latch onto genuine sui generis classes; or perhaps identify homeostatic property clusters; or even pick out no stably occurring distinctive features at all, elucidating important questions in the metaphysics of implicit attitudes.

## Footnotes

1 For instance, participants are faster to pair racialised-as-white names with positive terms than they are to pair racialised-as-black names with positive terms, (Greenwald et al. 1998), and faster to pair black faces with negative terms than with positive terms, (Dovidio et al. 1997). Participants are also faster at responding on stereotype congruent trials when matching gender and career concepts, gender and science concepts, and age and valenced concepts (Nosek et al. 2005) as well as sexuality and valenced concepts (Dasgupta and Rivera 2006) and disability and valenced concepts, (Lane et al. 2007).

2 For subtly differential treatment in social interactions see Chen and Bargh 1997; Dovidio et al. 1997; McConnell and Leibold 2001; Wilson *et al.* 2000; and for preferential treatment in more deliberative scenarios see Uhlmann and Cohen 2005; Green et al. 2007; Rooth 2007.

3 There has been much recent discussion of exactly how much behaviour the IAT predicts, with Oswald et al. claiming that it is a “poor” predictor of discriminatory behaviour (2013). However, as Levy points out, even on Oswald et al.’s account, this claim is false (Levy 2015; 803). Here, the authors indicate that the IAT reliably predicts about 2.5% of the variance in discriminatory behaviour. Discovering small, significant effects is importantly different to discovering non-significant effects, particularly with respect to their cumulative influence over the lifetime of an already marginalized person. Further, the behaviour in question can have a pronounced impact on a person’s life (such as prescribing medication; hiring a candidate; or determining whether to shoot someone).

4 I thank an anonymous referee for raising the possibility that implicit attitudes instantiate a homeostatic property cluster, and for pressing me to consider how this possibility interacts with the greater project of answering questions 1–4.

5 Gawronski and Bodenhausen claim that in order to form a new proposition in this manner, the considered information must still “pass a process of propositional validation” which “involves the acquisition of self-generated propositional information,” (2014, 453) by which I take them to mean that a person *infers* a new proposition from their previous experiences of the co-occurrence of two concepts. For instance, a person might see multiple instances of trees with green leaves, and infer, on the basis of these experiences, the proposition “trees are green.” According to Gawronski and Bodenhausen, this process of inference does not happen in the case of the formation of an association, and so for them, even if not necessarily for de Houwer (2014), the process of proposition formation is importantly distinct from association formation.

6 Note that application of these techniques does not guarantee that the associations in question *will* be extinguished or modified (Gawronski and Bodenhausen 2014, 454).

7 Levy points out that whilst the Gawronski et al. (2005) study is evidence of inferential processing, it is not obvious from the experimental paradigm that this occurs over *implicit* attitudes, rather than explicit ones. He maintains that because the stimuli in the Gawronski et al. (2005) were presented consciously, it is possible that participants could have formed explicit attitudes about the CS2s that exemplify ‘enemy-of-my-enemy-is-my-friend’ logic, and that these explicit attitudes were what experimenters measured. In defense of the use of this study, it is worth pointing out that Gawronski et al. (2005) tested the attitudes in question with an implicit measure: the affective priming task. According to psychologists, attitudes measured on this type of test have at least implicit feature *c* (as discussed in the introduction) in that they are activated automatically, and cannot be intentionally inhibited, which tags them as at least partly implicit (Payne and Gawronski 2010).

8 I thank an anonymous referee for encouraging me to clarify my response here.

9 Begby (2013) suggests that if a prejudice is internalised, then it may mediate the acceptance of new evidence, leading the person interpret putative counter evidence to their prejudiced belief as an exception to the rule. If so, Soloman might then count as responding to (what he takes to be) evidence. But this response is unlikely to work when counter-evidence is experienced from many domains: examples of women politicians, journalists, CEOs, etc., capable of abstract thought, can only be interpreted as exceptions to the rule up to a point. I am willing to countenance that, at least sometimes, explicitly prejudiced people have ample counter-evidence to their prejudices, not all of which may be interpreted as exceptions-to-the-rule, and still they fail to update their prejudiced belief accordingly. Some cases of prejudice remain highly resistant to evidence.
